# Comparison of CT- and MRI-Based Quantification of Tumor Heterogeneity and Vascularity for Correlations with Prognostic Biomarkers and Survival Outcomes: A Single-Center Prospective Cohort Study

**DOI:** 10.3390/bioengineering10050504

**Published:** 2023-04-22

**Authors:** Hyo-Young Kim, Min-Sun Bae, Bo-Kyoung Seo, Ji-Young Lee, Kyu-Ran Cho, Ok-Hee Woo, Sung-Eun Song, Jaehyung Cha

**Affiliations:** 1 Department of Radiology, Korea University Ansan Hospital, Korea University College of Medicine, 123 Jeokgeum-ro, Danwon-gu, Ansan City 15355, Republic of Korea; giantrabbit328@korea.ac.kr; 2 Department of Radiology, Inha University Hospital, Inha University College of Medicine, Inhang-ro 27, Jung-gu, Incheon 22332, Republic of Korea; msbae@inha.ac.kr; 3 Department of Radiology, Ilsan Paik Hospital, Inje University College of Medicine, 170 Juhwa-ro, Ilsanseo-gu, Goyang 10380, Republic of Korea; drleeji@paik.ac.kr; 4 Department of Radiology, Korea University Anam Hospital, Korea University College of Medicine, 73 Goryeodae-ro, Seongbuk-gu, Seoul 02841, Republic of Korea; krcho@korea.ac.kr (K.-R.C.);; 5 Department of Radiology, Korea University Guro Hospital, Korea University College of Medicine, 148 Gurodong-ro, Guro-gu, Seoul 08308, Republic of Korea; wokhee@korea.ac.kr; 6 Medical Science Research Center, Korea University Ansan Hospital, Korea University College of Medicine, 123 Jeokgeum-ro, Danwon-gu, Ansan City 15355, Republic of Korea; dolcha@korea.ac.kr

**Keywords:** breast neoplasms, quantitative evaluation, computed tomography, magnetic resonance imaging, survival, histogram analysis, perfusion analysis

## Abstract

Background: Tumor heterogeneity and vascularity can be noninvasively quantified using histogram and perfusion analyses on computed tomography (CT) and magnetic resonance imaging (MRI). We compared the association of histogram and perfusion features with histological prognostic factors and progression-free survival (PFS) in breast cancer patients on low-dose CT and MRI. Methods: This prospective study enrolled 147 women diagnosed with invasive breast cancer who simultaneously underwent contrast-enhanced MRI and CT before treatment. We extracted histogram and perfusion parameters from each tumor on MRI and CT, assessed associations between imaging features and histological biomarkers, and estimated PFS using the Kaplan–Meier analysis. Results: Out of 54 histogram and perfusion parameters, entropy on T2- and postcontrast T1-weighted MRI and postcontrast CT, and perfusion (blood flow) on CT were significantly associated with the status of subtypes, hormone receptors, and human epidermal growth factor receptor 2 (*p* < 0.05). Patients with high entropy on postcontrast CT showed worse PFS than patients with low entropy (*p* = 0.053) and high entropy on postcontrast CT negatively affected PFS in the Ki67-positive group (*p* = 0.046). Conclusions: Low-dose CT histogram and perfusion analysis were comparable to MRI, and the entropy of postcontrast CT could be a feasible parameter to predict PFS in breast cancer patients.

## 1. Introduction

Increased heterogeneity and vascularity of the tumor are poor prognostic factors for breast cancer. The heterogeneous nature of tumors is manifested at gross, cellular, and genetic levels because various mutations occur during tumor development [1]. Tumor heterogeneity limits targeted therapies and increases treatment resistance [2]. Angiogenesis is a process of new vessel formation that supplies oxygen and nutrients for tumor growth and promotes metastasis [3,4]. Therefore, the investigation of tumor heterogeneity and vascularity is necessary to evaluate treatment response, predict prognosis, and establish a treatment strategy tailored to each cancer patient. Tumor heterogeneity and vascularity can be evaluated histologically by tissue biopsy before treatment planning. However, a biopsy is only a partial sample of a cancer, making it difficult to capture the characteristics of the entire tumor. In addition, a biopsy is an invasive method that makes repeated treatment evaluation examinations uncomfortable, difficult, or even impossible.

Many studies have shown that quantification tools using histogram and perfusion analysis in magnetic resonance imaging (MRI) or computed tomography (CT) are useful for noninvasively measuring tumor heterogeneity and vascularity in breast cancer in prospective and retrospective cohorts [5,6,7,8,9,10]. In those studies, the histogram and perfusion parameters are associated with prognostic factors or responses to neoadjuvant chemotherapy. However, radiation exposure has limited the use of CT in breast cancer [11,12]. The breast is a radiation-sensitive organ and should be examined at a low radiation dose. However, as the radiation dose is reduced, the image quality deteriorates. Therefore, a CT scan with the lowest possible radiation dose is needed in order to maintain optimal image quality. Park et al. [13] demonstrated the potential of low-dose perfusion CT for quantifying vascularity in breast cancer with a significantly low radiation dose (an effective dose of 1.30–1.40 mSv for each patient) and the correlations of perfusion parameters on CT with histological prognostic factors and MRI kinetic characteristics. Since then, recent studies using low-dose perfusion CT or conventional chest CT demonstrated that quantitative histogram and perfusion parameters perform well in predicting prognostic factors, survival outcomes, and treatment failure rate in breast cancer [10,14,15,16]. Compared with MRI, CT has the advantages of a quick scan time, less discomfort during examination, and the ability to evaluate extensive lymph node enlargement, including mediastinum, lower neck, or internal mammary area, and distant metastases in the lungs and bony thorax. Therefore, CT can be useful in patients with advanced breast cancer or patients who are difficult to examine by MRI. Typical contraindications for breast MRI include allergy to gadolinium-based contrast media, implantable devices, severe obesity, and inability to undergo long examination (such as severe claustrophobia, inability to lie prone, or marked spinal deformity) [17].

To date, few comparative studies between CT and MRI have used the histogram and perfusion quantitative characteristics of breast cancer to evaluate their association with prognostic factors or survival. Here, we hypothesized that the quantitative tumor heterogeneity and vascularity parameters captured by low-dose CT would be comparable to those of MRI in association with histological biomarkers and survival outcomes and that CT could be an alternative in patients for whom it is difficult to perform MRI.

The aim of this study is to compare the association of histogram and perfusion analyses with histological prognostic biomarkers and progression-free survival (PFS) outcomes in breast cancer patients on low-dose CT and MRI.

## 2. Materials and Methods

### 2.1. Patients

**Figure 1:**
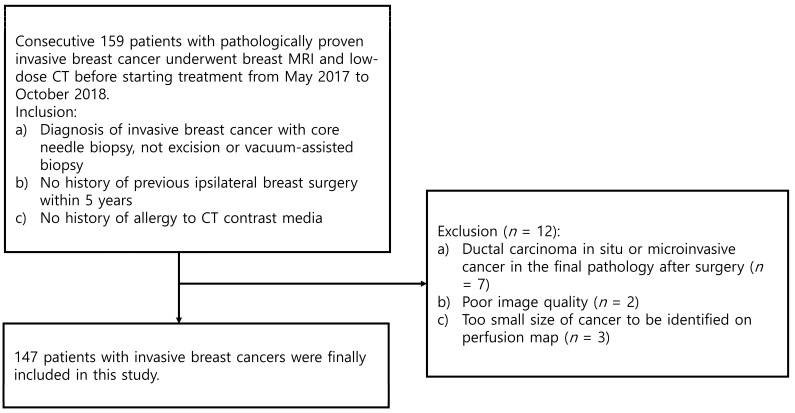
Flowchart of the study population.

The Institutional Review Board of Korea University Ansan Hospital approved this prospective study. Written informed consent was received from all participants. Consecutively, 159 women with pathologically proven invasive breast carcinomas underwent dynamic contrast-enhanced breast MRI and low-dose breast CT before starting treatment from May 2017 to October 2018. These participants were included in two previous prospective studies to evaluate the usefulness of histogram and perfusion analyses on CT or MRI used to predict prognostic factors in breast cancer [14,18]. In the current study, we compared the associations between image-analyzing parameters and histological biomarkers and survival outcomes of breast cancers on low-dose CT and MRI. Inclusion criteria for this study were as follows: (a) patients diagnosed pathologically with invasive breast carcinomas with core needle biopsy, not excision or vacuum-assisted biopsy; (b) patients without previous ipsilateral breast surgery within the last five years; and (c) patients with no history of allergic reaction to CT contrast media. Twelve patients were excluded because of ductal carcinoma in situ or microinvasive cancer in the final pathologic report after the operation (*n* = 7), image quality degradation due to motion artifact (*n* = 2), or cancer of too small a size to be identified on perfusion map (*n* = 3). Finally, 147 women (mean age 52.3 years, age range 25–81 years) with invasive breast carcinomas were included in our study. Figure 1 summarizes the study population, and Table 1 shows the tumor characteristics. Tumor sizes on MRI ranged from 6 to 109 mm (mean size ± standard deviation 23.9 ± 14.8 mm).

### 2.2. MRI Analysis

Two radiologists (J.Y.L. and B.K.S., with 10 and 21 years of experience in breast imaging, respectively) evaluated MRI and CT images and reached a consensus. They were blinded to the clinical and histological information. If a patient had simultaneous bilateral cancers, the largest tumor was selected.

MRI was performed in the prone position using a dedicated 4-channel breast coil on a 3.0 T MRI system (MAGNETOM Skyra; Siemens Healthineers, Erlangen, Germany). MRI was conducted according to a previous study [18]. Bilateral axial T2-weighted two-dimensional turbo spin-echo imaging with fat saturation (repetition time [ms] 4050, echo time [ms] 56, field of view [mm] 340 × 340, matrix [pixel] 307 × 384, flip angle [°] 120, reconstruction voxel size [mm] 0.44 × 0.44 × 3, slice thickness [mm] 3) and T1-weighted three-dimensional volumetric interpolated breath–hold imaging with fat saturation (repetition time 3.44, echo time 1.36, field of view 320 × 320, matrix 320 × 320, reconstruction voxel size 1 × 1 × 1, slice thickness 1) were obtained. Precontrast T1 mapping was generated using two different flip angles (2°, 9°) in the axial plane encompassing the whole tumor volume before dynamic examination. Postcontrast images were acquired after gadoterate meglumine (Uniray; Dongkook Life Science Co. Ltd., Seoul, Republic of Korea) was injected intravenously at a dose of 0.2 mL/kg of body weight, followed by a 30 mL saline flush. One precontrast image and five postcontrast images were obtained for dynamic examination. Postcontrast images were acquired at 93 s, 180 s, 268 s, 356 s, and 443 s after the injection of contrast media. T2-weighted images (T2), T1-weighted images before contrast media injection (PrecontrastT1), and T1-weighted images scanned at the first phase—93 s after contrast injection—(PostcontrastT1) were used for histogram analysis. Histogram features were analyzed using commercial software (TexRAD; Feedback Medical Ltd., London, UK), which is a first-order statistical-based histogram analysis technique [8,10,14,18]. Perfusion features were analyzed using a commercial algorithm (Tissue 4D; Siemens Healthineers) with Toft’s model implementation. [5,19,20]. On MRI, 18 histogram parameters and 16 perfusion parameters were obtained from each tumor.

**Figure 2:**
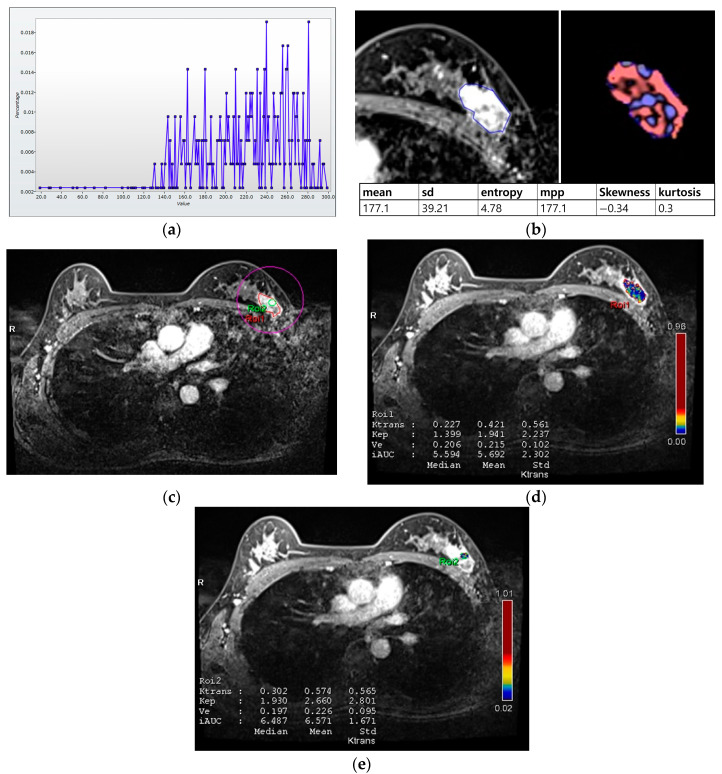
Histogram and perfusion analyses conducted on breast MRI in a 42-year-old woman with a 30 mm triple-negative invasive ductal carcinoma of the left breast (**a**,**b**). Histogram analysis on MRI: Axial contrast-enhanced T1-weighted MRI image shows an oval shaped, irregular marginated, and heterogeneous enhancing mass in the left breast. The region of interest (ROI) was drawn manually for the entire tumor, and a histogram was obtained (**a**). From the histogram, six statistically based metrics were extracted: mean, standard deviation (SD), mean of positive pixels (MPP), entropy, skewness, and kurtosis (**b**). (**c**–**e**) Perfusion analysis on MRI: Two ROIs (ROI1: entire tumor and ROI2: hot spot) were manually delineated (**c**), and eight perfusion parameters from each ROI were obtained: median and mean values of Ktrans, kep, Ve, and iAUC (**d**,**e**).

For histogram analysis, a region of interest (ROI) was drawn along the entire tumor enhancement on PostcontrastT1 in the largest cross-sectional area of the tumor, and this was then used for PrecontrastT1 and T2 lesions corresponding to those on PostcontrastT1. After tumor segmentation, six parameters were extracted from each ROI without filtering: (a) mean pixel intensity, (b) standard deviation (variation from the mean), (c) means of positive pixels (the average gray level intensity above zero threshold), (d) entropy (the randomness of gray-level distribution), (e) kurtosis (the peakedness of the distribution), and (f) skewness (asymmetry of the distribution) (Figure 2a,b).

For perfusion analysis, measurements were made in two ROIs for the entire tumor and hot spot (high perfusion area within the tumor; Figure 2c–e). Perfusion parameters were calculated using voxel-wise T1 perfusion maps, gadolinium concentration–time, arterial course input function, and fitting with a pharmacokinetic model. Four perfusion parameters were extracted from two ROIs for each tumor: (a) K^trans^ (the constant representing the transfer of contrast medium from blood plasma to the extracellular extravascular space per minute), (b) k_ep_ (the rate constant representing transfer of contrast medium from the extracellular extravascular space into blood plasma per minute), (c) V_e_ (the extracellular extravascular space per unit volume of tissue), and (d) iAUC (the initial area under the contrast concentration–time) [18,21,22]. The hot spot was selected as the high perfusion area on the K^trans^-based perfusion map. The median and mean values of each parameter were automatically calculated from the two ROIs.

### 2.3. CT Analysis

Low-dose perfusion CT was performed according to a previous study [16]. CT was conducted using an IQon Spectral CT scanner (Philips Health Systems, Cleveland, OH, USA). An additional table pad with a rectangular hole was inserted for placing the breast on a normal CT table for examination in the prone position [13,16]. A radiologist (B.K.S) evaluated prior mammography, ultrasound, or MRI before the CT scans and performed a targeted ultrasound to localize the tumor. After localizing the cancer, a skin marker (X-spot; Beekley Medical, Bristol, CT, USA) was applied to the skin at the cancer site. Low-dose perfusion CT was performed as follows: tube voltage 80 kVp, tube current 25 mA or 30 mA, collimation 64 mm × 0.625 mm, rotation time 0.5 s, matrix 512 × 512, and slice thickness 5 mm. The scan range was 40 mm along the *z*-axis, including the skin markers. After setting the scan range on a precontrast scan, the skin marker was removed, and contrast media was injected. A total of 60 mL of Xenetix 350 (Guerbet, Aulnay-sous-Bois, France) was administered at a rate of 4 mL/s. Eighteen scans were performed at 3 s intervals after contrast media injection, and four scans at 30 s intervals (effective dose for each patient, 1.01–1.38 mSv). Histogram features on CT were analyzed using the same commercial software as MRI. Perfusion features were calculated with a maximum slope algorithm using a commercial software (Functional CT; Philips Health Systems). On CT, 12 histogram parameters and 8 perfusion parameters were obtained from each tumor.

**Figure 3:**
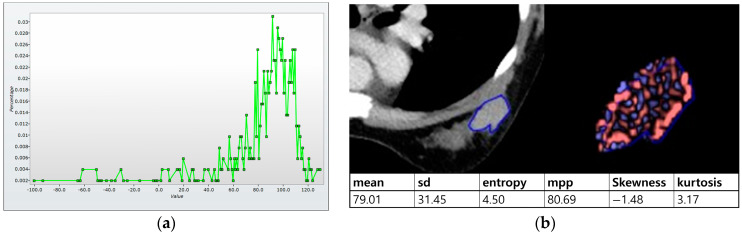

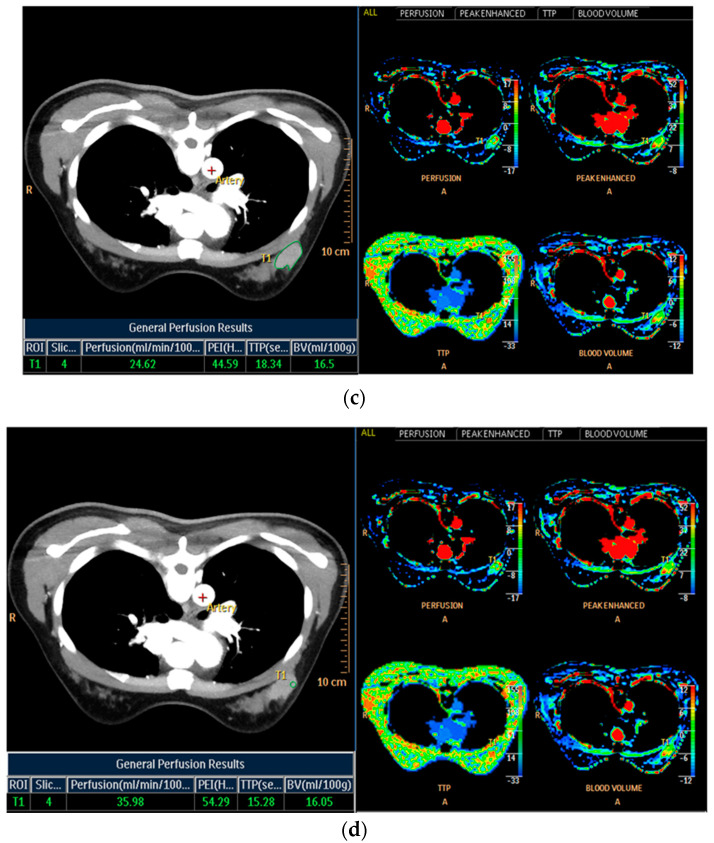
Histogram and perfusion analyses on breast CT in a 42-year-old woman with a 30 mm triple-negative invasive ductal carcinoma of the left breast (the same patient as Figure 2). (**a**,**b**) Histogram analysis on CT: Axial contrast-enhanced low-dose CT shows an oval shaped, irregular marginated, and heterogeneous enhancing mass. Histogram analysis on CT was performed using the same method on MRI. (**c**,**d**) Perfusion analysis on CT: Two ROIs (ROI1: entire tumor and ROI2: hot spot) were manually delineated and four perfusion parameters from each ROI were obtained, perfusion, peak enhancement intensity (PEI), time to peak (TTP), and blood volume (BV).

Histogram analysis on CT was performed on pre- and postcontrast images using the same method as on MRI (Figure 3a,b). When the tumor was maximally enhanced, an ROI containing the entire tumor was drawn [14]. In perfusion analysis on CT, similarity to MRI, two ROIs, total tumor area and hot spot were measured. In addition, time–intensity curves and perfusion color maps for the tumors were measured automatically when we drew ROIs. Four parameters on CT perfusion maps were measured from the two ROIs for each cancer: (a) perfusion (blood flow; mL/min per 100 mL), (b) blood volume (total blood volume over the region during the scan period; mL/100 g), (c) peak enhancement intensity (peak enhancement after contrast media injection; Hounsfield units [HU]), and (d) time to peak (time to reach peak contrast enhancement; seconds; Figure 3c,d).

### 2.4. Histological Evaluation

The histological reports were reviewed in order to evaluate the status of prognostic factors and molecular subtypes. After the CT and MRI examinations, surgery was performed in 129 (88%) patients, neoadjuvant chemotherapy plus surgery was performed in 11 (7%) patients, and chemotherapy was performed in 7 (5%) patients. Thus, histological findings were obtained from 129 surgical and 18 tissue biopsy specimens. We evaluated the status of estrogen receptor (ER), progesterone receptor (PR), human epidermal growth factor receptor 2 (HER2), Ki67, grade, and subtype, and dichotomized their results for statistical analysis, i.e., ER, PR, HER2, and Ki67 status were divided into positive or negative. The Allred scoring system defined ER and PR positivity as a score of 3 or greater [23]. HER2 positivity was defined when the tumor had 2+ for immunohistochemical staining plus HER2 gene amplification in silver-stained in situ hybridization or 3+ immunohistochemical staining [24]. Ki67 positivity was defined when the expression was greater than 20% [25]. The grade was divided into low (1 or 2) and high (3) [26,27]. Molecular subtypes were divided into luminal A (ER and/or PR positive, HER2 negative, and Ki67 negative), luminal B (ER and/or PR positive and HER2 positive; or ER and/or PR positive, HER2 negative, and Ki67 positive), HER2-enriched (ER negative, PR negative, and HER2 positive), and triple-negative cancer (ER negative, PR negative, and HER2 negative) [28].

### 2.5. Statistical Analysis

The Mann–Whitney *U*-test or *t*-test was used to compare the associations between MRI and CT imaging phenotype and dichotomized histologic biomarker groups such as ER, PR, HER2, Ki67, and grade. To evaluate the association between imaging features and molecular subgroups, the Kruskal–Wallis test and analysis of variance followed by a post hoc test were used to identify the different subgroups. A *p*-value < 0.003 (0.05/18) for MRI histogram parameters, a *p*-value < 0.003 (0.05/16) for MRI perfusion parameters, a *p*-value < 0.004 (0.05/12) for CT histogram parameters, and a *p*-value < 0.006 (0.05/8) for CT perfusion parameters were used to decide significance. For multiple comparisons, *p*-values were adjusted using the Bonferroni correction.

PFS was defined from the date of histologic diagnosis until the date of the first observation of documented disease relapse, progression, or death. For patients without progression, PFS was defined as the date of the last follow-up [29]. Disease progression was defined as locoregional recurrence, new primary contralateral breast cancer, and distant metastasis after treatment completion, except for patients with distant metastases or contralateral breast cancer at diagnosis [30]. We confirmed locoregional recurrence by tissue biopsy and metastasis by either tissue biopsy or imaging studies [30,31]. The Kaplan–Meier analysis was performed to estimate PFS, and the results were compared using the log-rank test. Statistical analyses were performed using IBM SPSS Statistics (version 25.0, IBM Corp., Armonk, NY, USA) and Python 3.52 in December 2022.

## 3. Results

### 3.1. Associations between MRI and Histological Biomarkers

**Figure 4:**
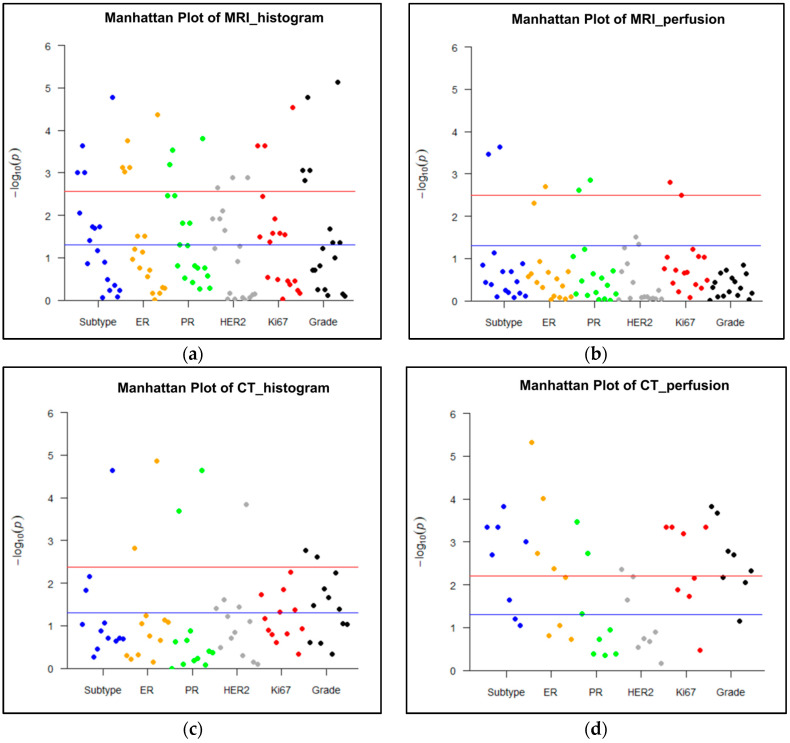
Manhattan plots representing the association between MRI or CT parameters and histological biomarkers. A *p* value < 0.003 (0.05/18 parameters) for MRI histogram features, a *p* value < 0.003 (0.05/16 parameters) for MRI perfusion features, a *p* value of < 0.004 (0.05/12 parameters) for CT histogram features, and a *p* value < 0.006 (0.05/8 parameters) for CT perfusion features were used to decide significance using Bonferroni correction for multiple comparisons. A blue line represents *p* < 0.05, and red line represents corrected *p* values after Bonferroni correction for each imaging analysis. (**a**) Histogram analysis on MRI: Histogram parameters were significantly associated with all histological findings, such as molecular subtype, expression of estrogen receptor (ER), progesterone receptor (PR), human epidermal growth factor receptor 2 (HER2), and Ki67, and grade (*p* < 0.003). (**b**) Perfusion analysis on MRI: Perfusion parameters were significantly correlated with the status of subtype, ER, PR, and Ki67 (*p* < 0.003). (**c**) Histogram analysis on CT: Histogram parameters were significantly associated with all histological biomarkers except for Ki67 expression (*p* < 0.004). (**d**) Perfusion analysis on CT: Perfusion parameters were significantly correlated with all histological findings (*p* < 0.006).

The associations between MRI histogram parameters and histological findings are shown in Table 2 and Figure 4a. Entropy on PostcontrastT1 was significantly associated with all histological factors, namely subtype, ER, PR, HER2, Ki67, and grade (*p* < 0.003). Entropy on the T2 image was significantly correlated with all histological biomarkers except for Ki67 (*p* < 0.003). Entropy on Postcontrast T1 and T2 was higher in cancers with poor prognostic factors such as ER negativity, PR negativity, HER2 positivity, Ki67 positivity, high grade, and nonluminal subtypes including HER2-enriched and triple-negative cancers (Table 3). The mean value, standard deviation, and mean of positive pixels on T2 were significantly correlated with the status of subtype, ER, PR, Ki67, or grade (*p* < 0.003). Entropy on PrecontrastT1 were correlated with the status of HER2 (*p* < 0.003).

The associations between MRI perfusion parameters and histological factors are shown in Table 4 and Figure 4b. The median and mean V_e_ of the entire tumor were significantly correlated with the status of the subtype, ER, PR, or Ki67 (*p* < 0.003). The other tumor and hot spot perfusion parameters were not significantly correlated with any histological biomarkers.

### 3.2. Associations between CT and Histological Biomarkers

The entropy values on postcontrast CT were higher in cancers with poor prognostic factors such as ER negativity, PR negativity, HER2 positivity, Ki67 positivity, high grade, and nonluminal subtypes, including HER2-enriched and triple-negative cancers (Table 3). These differences were statistically significant according to the status of subtype, ER, PR, and HER2 (*p* < 0.004). The associations between CT histogram features and histological factors are shown in Table 5 and Figure 3c. Other histogram parameters on postcontrast images did not show any correlations with histological biomarkers. On precontrast CT images, entropy, mean, and skewness correlated with the status of ER, PR, or grade (*p* < 0.004).

The associations between CT perfusion features and histological biomarkers are shown in Table 6 and Figure 3d. The perfusion of hot spots was associated with all histological factors (*p* < 0.006). The peak enhancement intensity, the time to peak, and the blood volume obtained from hot spots were correlated with subtype, ER, PR, Ki67, or grade. The perfusion and the blood volume measured from the entire tumor were significantly associated with subtype, ER status, Ki67 status, or grade (*p* < 0.006). The time to peak and the peak enhancement intensity of the entire tumor did not show any correlations with histological biomarkers.

### 3.3. Progression-Free Survival

**Figure 5:**
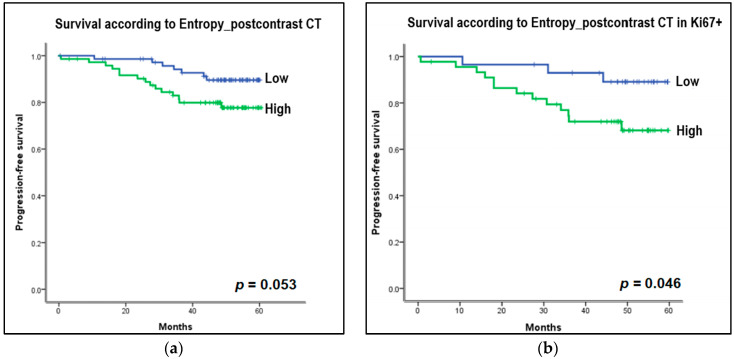

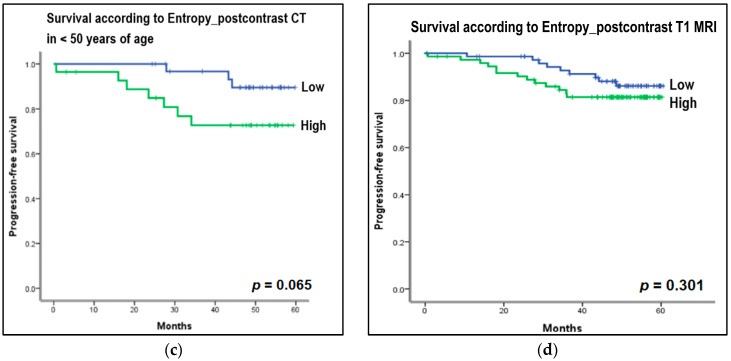
Kaplan–Meier curves to estimate progression-free survival (PFS) according to the entropy of histogram parameters on CT and MRI. For survival analysis, the collected CT and MRI histogram parameter data were dichotomized based on median value. (**a**) Entropy on postcontrast CT: High entropy group (>4.62 Hounsfield unit [HU]) on postcontrast CT images showed decreased PFS compared to the low entropy group (≤4.62 HU) on postcontrast CT images (*p* = 0.053). (**b**) Entropy on postcontrast CT in Ki67-positive group: In the subgroup analysis, in Ki67-positive patients, the group with high postcontrast CT entropy (>5.10 HU) showed a statistically significant decrease in PFS compared to the group with low postcontrast CT entropy (≤5.10 HU) (*p* = 0.046). (**c**) Entropy on postcontrast CT in the younger age group, less than 50 years: In the subgroup analysis, the high postcontrast CT entropy group (>5.06 HU) showed a reduced PFS in the younger age group (age <50 years), but this was not statistically significant (≤5.05 HU) (*p* = 0.065). (**d**) Entropy on postcontrast T1-weighted MRI: The high entropy group (>4.95) on postcontrast T1-weighted MRI showed a decrease in PFS compared to the low entropy group (≤4.95), but this was not statistically significant (*p* = 0.301).

The median follow-up time was 57 months (range, 0–67 months). Events occurred in 23 patients out of a total of 147 patients: five deaths, nine locoregional recurrences, seven new distant metastases, one disease progression, and one new contralateral breast cancer. For survival analysis, the collected CT and MRI histogram and perfusion parameter data were dichotomized based on the median value and were evaluated with the Kaplan–Meier curve. Among the CT histogram and perfusion parameters, the high-entropy group (>4.62 HU) on postcontrast CT images showed decreased PFS compared with the low-entropy group (≤4.62 HU) on postcontrast CT images (*p* = 0.053) (Figure 5a). In the subgroup analysis, in Ki67-positive patients, the group with high-postcontrast CT entropy (>5.10 HU) showed a statistically significant decrease in PFS compared with the group with low postcontrast CT entropy (≤5.10 HU) (*p* = 0.046; Figure 5b). Furthermore, the high-postcontrast CT entropy group (>5.05 HU) showed a reduced PFS in the younger age group (age < 50 years), but this difference was not statistically significant (≤5.05 HU; *p* = 0.065; Figure 5c). Other factors such as the status of ER, PR, and HER2 (positive vs negative), tumor grade (1, 2 vs 3), and subtype (luminal vs nonluminal) showed no difference in PFS between the subgroups. Among MRI histogram and perfusion parameters, the high-entropy group (>4.95) on postcontrast T1-weighted MRI showed a decrease in PFS compared with the low-entropy group (≤4.95), but this was not statistically significant (*p* = 0.301; Figure 5d).

## 4. Discussion

Our study showed that noninvasive quantification of tumor heterogeneity and vascularity are associated with histological prognostic factors, namely, molecular subtype, ER, PR, HER2, Ki67, and grade, and MRI and CT of invasive breast cancer. Of the MRI parameters, the entropy on PostcontrastT1 was significantly correlated with all prognostic biomarkers, and the subtype and entropy on T2 were associated with all biomarkers except for Ki67. Of the CT parameters, the perfusion of hot spots correlated with all prognostic factors and subtypes, and the entropy on postcontrast images was associated with subtypes, ER, PR, and HER2. Notably, the entropy on postcontrast CT images was related to survival outcome, and the group with high-postcontrast CT entropy showed a significant decrease in PFS in the Ki67-positive group.

Based on our study, entropy was the most valuable quantitative imaging parameter for the prediction of prognostic biomarkers in patients with invasive breast cancer on MRI and CT. Entropy shows the randomness of the gray-level distribution of a histogram in a given ROI [32]. Our results are consistent with previous studies. Since entropy is higher in malignant breast lesions than in benign lesions, it is useful for differentiating benign breast lesions from malignant ones [33,34]. In breast cancers, entropy was significantly different according to the subtypes and prognostic histological factors and increased in aggressive cancers [18,35]. In addition, we demonstrated in this study that the entropy of postcontrast CT was related to PFS. In the subgroup analysis, the high-entropy group on postcontrast CT had a negative effect on PFS in the Ki67-positive group and the younger age group (under 50 years of age). It is already known that Ki67 positivity and young age are indicators of poor prognosis and poor response to treatment in breast cancer [36,37,38,39,40,41]. This finding could imply that entropy on CT images after contrast injection could be promising as an imaging biomarker in precision medicine and useful for treatment planning and posttreatment surveillance monitoring of high-risk breast cancer patients. Chamming’s et al. [8] reported that kurtosis of histogram parameters on MRI was related to ER and tumor grade, and kurtosis in particular showed good performance in identifying triple-negative cancer. The authors also demonstrated that kurtosis on contrast-enhanced T1-weighed MRI is an important histogram feature to predict complete pathological response to neoadjuvant chemotherapy in breast cancer. However, in this study, kurtosis was not related to histological prognostic biomarkers and subtypes.

Considering the perfusion parameters, the perfusion value of the hot spot on CT shows correlations with all histological factors, and the V_e_ of the entire tumor on MRI was associated with subtype, ER, PR, and Ki67. Perfusion on CT measures blood flow through the vasculature in a defined tumor volume [13,42,43]. Tumor angiogenesis refers to the formation of new vessels, the development of arteriovenous shunts, and hyperpermeability, which increases the volume and rate of blood flow. Therefore, increased perfusion value on CT could be associated with increased angiogenesis and aggressive tumor. Perfusion CT for oncology is valuable for staging, predicting prognosis, and evaluating tumor response to chemo- or radiation therapy. However, the analysis of perfusion CT has not been standardized in various organs [44]. In the breast, perfusion (blood flow) and blood volume are significantly correlated with microvessel density in the tumor area in murine breast cancer in rats. These parameters were associated with prognostic histological factors in human studies [13,16,45]. In this study, we quantified perfusion, blood volume, time to peak, and peak enhancement intensity. These were associated with various prognostic biomarkers, and the results were consistent with previous perfusion CT studies [13,16]. V_e_ on MRI measures the volume fraction of extracellular extravascular space per unit of tissue volume. Thus, V_e_ is associated with tumor cellularity and viable tumor portions. V_e_ has been shown to decrease in breast cancers with ER negativity and Ki67 positivity in previous studies, and our results were consistent with previous MRI perfusion studies [22,46,47]. In addition, Nagasaka et al. [46] reported that the variation in Ve was greater in cancers with ER negativity and Ki67 positivity compared to cancers with ER positivity and Ki67 negativity on histogram analysis of quantitative perfusion MRI parameters. Therefore, both Ve value and variation in Ve within tumors may be associated with poor prognostic factors in breast cancer. Except for Ve, other MRI perfusion parameters—K^trans^, K_ep_, and iAUC—were not associated with prognostic biomarkers in this study, so the number of points showing statistically significant correlations with histological biomarkers was low in the Manhattan plot of MRI perfusion when compared to the plots of MRI histogram, CT histogram, and CT perfusion. Quantitative perfusion parameters on CT and MRI were found to be associated with histological prognostic factors in breast cancer but were not associated with PFS in this study.

This study suggests three advances in quantifying medical imaging in breast cancer. First, in a prospective cohort, we compared the associations of prognostic biomarkers and quantitative histogram and perfusion parameters between CT and MRI in patients who underwent both imaging modalities concomitantly. Few comparative studies between CT and MRI have used quantitative imaging features of breast cancer to evaluate their association with prognostic factors or survival outcomes. CT has advantages in terms of oncology imaging and quantifying medical images of breast cancer. CT can evaluate the lungs, bony thorax, mediastinum, lymph nodes, and breast. It consumes much less energy than MRI [48] and provides absolute pixel intensity values and Hounsfield units. Second, commercial software was used to quantify breast cancer heterogeneity and vascularity in this study, and the results may be applied to multicenter studies. Third, we used a low-dose perfusion CT protocol. The effective dose ranged from 1.30 mSv to 1.40 mSv for each patient. The average effective dose for acceptable low-dose chest CT screening is approximately 2 mSv, which is very low compared to a standard-dose chest CT [49]. Given that the average annual effective dose from natural background radiation in the United States is about 3 mSv [50,51], our CT protocol used very low radiation doses.

Despite our best efforts, our study has several limitations. First, this prospective study was conducted with only a small number of patients in a single institution. Further efforts are needed to include different patient cohorts from several institutions and evaluate the clinical utility of these results. Second, low-dose CT was performed using a perfusion protocol, and CT scans were taken over a 4 cm range along the z-axis. Therefore, this approach could not cover the full extent of large tumors. Instead, we examined the center of large cancers measuring >40 mm. In the near future, advances in CT technology will allow the perfusion scan range to be extended while maintaining low radiation doses. Third, image standardization or non-uniform artifact correction before histogram analysis can improve reliability and reproducibility [32,52]. We did not perform image standardization or non-uniform correction before histogram analysis in this single-center preliminary study. However, a previous study showed that the use of commercial software (TexRAD) for histogram analysis in this study shows excellent inter- and intra-reader agreement for segmentation and Pearson correlation between each software pair [53]. Many studies used the same histogram analysis software in breast cancer and other body cancers, and these studies show correlations with prognostic factors or treatment responses [8,18,54]. For widespread clinical implementation of histogram analysis, standardization of segmentation, preprocessing and postprocessing of MRI and CT images are required. We will conduct further studies using image normalization and preprocessing for image uniformity in a large population to validate the results and evaluate clinical applications. Fourth, the histogram analysis software we used analyzes two-dimensional images, so the data may not fully reflect the textural features of the entire tumor in three dimensions. However, Lubner et al. [55] demonstrated that the histogram analysis results of two-dimensional and three-dimensional images are similar. Fifth, segmentation reproducibility was not assessed in this study. We manually drew ROIs to quantify perfusion and histogram characteristics based on the consensus of two experienced radiologists. Generalizing our findings in the near future will require automatic lesion segmentation and inter-observer variability.

## 5. Conclusions

In this prospective study, we found that data quantifying tumor heterogeneity and vascularity in invasive breast cancers using histogram and perfusion analysis algorithms correlated with molecular subtypes and histological prognostic biomarkers in MRI and CT images. Notably, high entropy (the randomness of the gray-level distribution) on postcontrast CT was associated with PFS in breast cancer, and high entropy on postcontrast CT images negatively affected PFS in the Ki67-positive group. Therefore, low-dose CT histogram and perfusion analysis were comparable to MRI. The entropy on postcontrast CT could be a feasible imaging parameter for the prediction of survival outcomes in breast cancer patients. However, since this prospective study was conducted with a small number of patients from a single institution, further studies with larger populations from diverse institutions are needed to verify our results and evaluate their usefulness in clinical practice.

## Figures and Tables

**Table 1:** Tumor characteristics.

Characteristic	No. of Breast Cancers (*n* = 147)
ER	
Negative	48 (33%)
Positive	99 (67%)
PR	
Negative	50 (34%)
Positive	97 (66%)
HER2	
Negative	123 (84%)
Positive	24 (16%)
Ki67	
Negative	73 (50%)
Positive	74 (50%)
Tumor grade	
Low	96 (65%)
High	51 (35%)
Molecular subtype	
Luminal A	65 (44%)
Luminal B	38 (26%)
HER2-enriched	18 (12%)
Triple-negative	26 (18%)

ER: estrogen receptor; PR: progesterone receptor; HER2: human epidermal growth factor receptor 2.

**Table 2:** *p* values for association between MRI histogram features and histological biomarkers.

Histogram Feature	Subtype	ER	PR	HER2	Ki67	Grade
Mean on T2	0.001	0.001	0.003	0.012	<0.001	0.001
Standard deviation on T2	0.009	0.001	0.001	0.060	0.032	0.002
Mean of positive pixels on T2	0.001	0.001	0.003	0.012	<0.001	0.001
Entropy on T2	<0.001	<0.001	<0.001	0.002	0.004	<0.001
Kurtosis on T2	0.039	0.063	0.050	0.023	0.043	0.198
Skewness on T2	0.138	0.107	0.152	0.008	0.285	0.196
Mean on PrecontrastT1	0.019	0.031	0.016	0.950	0.026	0.561
Standard deviation on PrecontrastT1	0.020	0.173	0.301	0.692	0.012	0.156
Mean of positive pixels on PrecontrastT1	0.019	0.031	0.016	0.950	0.026	0.561
Entropy on PrecontrastT1	0.069	0.073	0.052	0.001	0.323	0.061
Kurtosis on PrecontrastT1	0.127	0.196	0.155	0.054	0.028	0.021
Skewness on PrecontrastT1	0.853	0.275	0.377	0.123	0.946	0.770
Mean on PostcontrastT1	0.326	0.692	0.174	0.880	0.348	0.044
Standard deviation on PostcontrastT1	0.592	0.977	0.548	0.981	0.432	0.101
Mean of positive pixels on PostcontrastT1	0.442	0.692	0.174	0.880	0.348	0.044
Entropy on PostcontrastT1	<0.001	<0.001	<0.001	<0.001	<0.001	<0.001
Kurtosis on PrecontrastT1	0.598	0.523	0.514	0.714	0.683	0.801
Skewness on PostcontrastT1	0.824	0.502	0.266	0.740	0.598	0.702

ER: estrogen receptor; PR: progesterone receptor; HER2: human epidermal growth factor receptor 2; T2: T2-weighted images; PrecontrastT1: T1-weighted images before contrast media injection; and PostcontrastT1: T1-weighted images obtained in the first phase after contrast injection. A *p* value < 0.003 (0.05/18 parameters) was considered statistically significant.

**Table 3:** MRI and CT entropy values according to histological biomarker status.

Histological Biomarker	PostcontrastT1 MRI	T2 MRI	Postcontrast CT (HU)
Molecular subtype			
Luminal A	4.667 ± 0.491	4.775 ± 0.506	4.491 ± 0.337
Luminal B	4.996 ± 0.535	4.996 ± 0.455	4.577 ± 0.276
HER2-enriched	5.303 ± 0.384	5.327 ± 0.511	4.803 ± 0.212
Triple-negative	5.012 ± 0.347	5.069 ± 0.549	4.705 ± 0.218
ER			
Negative	5.133 ± 0.424	5.156 ± 0.577	4.734 ± 0.234
Positive	4.774 ± 0.516	4.853 ± 0.479	4.519 ± 0.316
PR			
Negative	5.092 ± 0.395	5.165 ± 0.537	4.734 ± 0.214
Positive	4.787 ± 0.540	4.842 ± 0.495	4.515 ± 0.323
HER2			
Negative	4.811 ± 0.505	4.887 ± 0.517	4.551 ± 0.312
Positive	5.300 ± 0.343	5.284 ± 0.477	4.788 ± 0.194
Ki67			
Negative	4.717 ± 0.509	4.829 ± 0.519	4.522 ± 0.348
Positive	5.063 ± 0.462	5.072 ± 0.517	4.656 ± 0.248
Tumor grade			
Low	4.763 ± 0.517	4.851 ± 0.511	4.542 ± 0.336
High	5.132 ± 0.418	5.141 ± 0.518	4.679 ± 0.225

HER2: human epidermal growth factor receptor 2; PostcontrastT1: T1-weighted images obtained in the first phase after contrast injection; T2: T2-weighted images; HU: Hounsfield unit.

**Table 4:** *p* values for the association between MRI perfusion features and histological biomarkers.

Perfusion Feature	Subtype	ER	PR	HER2	Ki67	Grade
Median K^trans^ of entire tumor	0.145	0.264	0.089	0.942	0.173	0.969
Median k_ep_ of entire tumor	0.364	0.227	0.675	0.208	0.092	0.488
Median V_e_ of entire tumor	<0.001	0.005	0.002	0.055	0.002	0.360
Median iAUC of entire tumor	0.412	0.361	0.343	0.131	0.385	0.804
Mean K^trans^ of entire tumor	0.074	0.117	0.061	0.883	0.187	0.219
Mean k_ep_ of entire tumor	0.789	0.484	0.731	0.365	0.610	0.784
Mean V_e_ of entire tumor	<0.001	0.002	0.001	0.030	0.003	0.187
Mean iAUC of entire tumor	0.204	0.209	0.225	0.046	0.218	0.605
Median K^trans^ of hot spot	0.573	0.924	0.635	0.836	0.212	0.291
Median k_ep_ of hot spot	0.629	0.768	0.940	0.803	0.845	0.346
Median V_e_ of hot spot	0.200	0.302	0.290	0.798	0.059	0.740
Median iAUC of hot spot	0.837	0.846	0.906	0.912	0.416	0.497
Mean K^trans^ of hot spot	0.353	0.444	0.422	0.863	0.090	0.146
Mean k_ep_ of hot spot	0.661	0.918	0.976	0.896	0.498	0.227
Mean V_e_ of hot spot	0.131	0.205	0.195	0.560	0.093	0.936
Mean iAUC of hot spot	0.763	0.798	0.674	0.912	0.329	0.660

ER: estrogen receptor; PR: progesterone receptor; HER2: human epidermal growth factor receptor 2; K^trans^: the constant representing the transfer of contrast medium from blood plasma to the extracellular extravascular space per minute; k_ep_: the rate constant representing the transfer of contrast medium from the extracellular extravascular space into blood plasma per minute; V_e_: the extracellular extravascular space per unit volume of tissue; iAUC: the initial area under the contrast concentration–time curve. A hot spot within the tumor was selected as an area of high perfusion on the K^trans^-based perfusion map. A *p* value < 0.003 (0.05/16 parameters) was considered statistically significant.

**Table 5:** *p* values for the association between CT histogram features and histological biomarkers.

Histogram Feature	Subtype	ER	PR	HER2	Ki67	Grade
Mean on precontrast CT	0.092	0.498	1.000	0.039	0.018	0.002
Standard deviation on precontrast CT	0.015	0.611	0.237	0.322	0.068	0.252
Mean of positive pixels on precontrast CT	0.541	0.477	0.806	0.060	0.163	0.002
Entropy on precontrast CT	0.007	0.002	<0.001	0.025	0.127	0.033
Kurtosis on precontrast CT	0.133	0.058	0.130	0.141	0.047	0.014
Skewness on precontrast CT	0.347	0.090	0.221	0.194	0.247	0.259
Mean on postcontrast CT	0.087	0.175	0.663	0.037	0.014	0.021
Standard deviation on postcontrast CT	0.199	0.721	0.585	0.492	0.153	0.473
Mean of positive pixels on postcontrast CT	0.226	0.218	0.832	0.080	0.042	0.041
Entropy on postcontrast CT	<0.001	<0.001	<0.001	<0.001	0.005	0.006
Kurtosis on postcontrast CT	0.203	0.082	0.426	0.801	0.118	0.092
Skewness on postcontrast CT	0.196	0.074	0.390	0.724	0.467	0.089

ER: estrogen receptor; PR: progesterone receptor; HER2: human epidermal growth factor receptor 2. A *p* value < 0.004 (0.05/12 parameters) was considered statistically significant.

**Table 6:** *p* values for the association between CT perfusion features and histological biomarkers.

Perfusion Feature	Subtype	ER	PR	HER2	Ki67	Grade
Perfusion of entire tumor	0.023	0.004	0.189	0.183	0.018	0.002
Peak enhancement intensity of entire tumor	0.062	0.090	0.442	0.212	0.007	0.070
Time to peak of entire tumor	0.088	0.007	0.112	0.127	0.340	0.009
Blood volume of entire tumor	0.001	0.189	0.407	0.690	<0.001	0.005
Perfusion of hot spot	<0.001	<0.001	<0.001	0.004	<0.001	<0.001
Peak enhancement intensity of hot spot	0.002	0.002	0.047	0.023	<0.001	<0.001
Time to peak of hot spot	<0.001	<0.001	0.002	0.006	0.013	0.007
Blood volume of hot spot	<0.001	0.157	0.408	0.287	0.001	0.002

ER: estrogen receptor; PR: progesterone receptor; HER2: human epidermal growth factor receptor 2. A *p* value < 0.006 (0.05/8 parameters) was considered statistically significant.

## Data Availability

The data presented in this study are available on request from the corresponding author. The data are not publicly available due to privacy.

## Abbreviations

CT	computed tomography
MRI	magnetic resonance imaging
PFS	progression-free survival
ROI	region of interest
T2	T2-weighted images
PrecontrastT1	T1-weighted images before contrast media injection
PostcontrastT1	T1-weighted images obtained in the first phase after contrast injection
K^trans^	the constant for the transfer of the contrast medium
k_ep_	the rate constant of the contrast medium
V_e_	the extracellular extravascular space
iAUC	the initial area under the contrast concentration–time curve
HU	Hounsfield units
ER	estrogen receptor
PR	progesterone receptor
HER2	human epidermal growth factor receptor 2
